# Brain caspase-3 and intestinal FABP responses in preterm and term rats submitted to birth asphyxia

**DOI:** 10.1590/1414-431X20165258

**Published:** 2016-06-23

**Authors:** R.L. Figueira, F.L. Gonçalves, A.L. Simões, C.A. Bernardino, L.S. Lopes, O. Castro e Silva, L. Sbragia

**Affiliations:** 1 Divisão de Cirurgia Pediátrica, Departamento de Cirurgia e Anatomia, Faculdade de Medicina de Ribeirão Preto, Universidade de São Paulo, Ribeirão Preto, SP, Brasil; 2 Neurocirurgia, Departamento de Cirurgia e Anatomia, Faculdade de Medicina de Ribeirão Preto, Universidade de São Paulo, Ribeirão Preto, SP, Brasil; 3 Divisão de Transplante, Departamento de Cirurgia e Anatomia, Faculdade de Medicina de Ribeirão Preto, Universidade de São Paulo, Ribeirão Preto, SP, Brasil

**Keywords:** Neonatal asphyxia, Necrotizing enterocolitis, I-FABP, Caspase-3, Brain injury, Intestinal damage

## Abstract

Neonatal asphyxia can cause irreversible injury of multiple organs resulting in hypoxic-ischemic encephalopathy and necrotizing enterocolitis (NEC). This injury is dependent on time, severity, and gestational age, once the preterm babies need ventilator support. Our aim was to assess the different brain and intestinal effects of ischemia and reperfusion in neonate rats after birth anoxia and mechanical ventilation. Preterm and term neonates were divided into 8 subgroups (n=12/group): 1) preterm control (PTC), 2) preterm ventilated (PTV), 3) preterm asphyxiated (PTA), 4) preterm asphyxiated and ventilated (PTAV), 5) term control (TC), 6) term ventilated (TV), 7) term asphyxiated (TA), and 8) term asphyxiated and ventilated (TAV). We measured body, brain, and intestine weights and respective ratios [(BW), (BrW), (IW), (BrW/BW) and (IW/BW)]. Histology analysis and damage grading were performed in the brain (cortex/hippocampus) and intestine (jejunum/ileum) tissues, as well as immunohistochemistry analysis for caspase-3 and intestinal fatty acid-binding protein (I-FABP). IW was lower in the TA than in the other terms (P<0.05), and the IW/BW ratio was lower in the TA than in the TAV (P<0.005). PTA, PTAV and TA presented high levels of brain damage. In histological intestinal analysis, PTAV and TAV had higher scores than the other groups. Caspase-3 was higher in PTAV (cortex) and TA (cortex/hippocampus) (P<0.005). I-FABP was higher in PTAV (P<0.005) and TA (ileum) (P<0.05). I-FABP expression was increased in PTAV subgroup (P<0.0001). Brain and intestinal responses in neonatal rats caused by neonatal asphyxia, with or without mechanical ventilation, varied with gestational age, with increased expression of caspase-3 and I-FABP biomarkers.

## Introduction

Considered to be a worldwide clinical problem, neonatal asphyxia (NA) is defined as the reduction of serum oxygen levels and nutrient supply to vital organs of neonates (1,2). Depending on its magnitude, NA can cause permanent neurological damage with or without mental deficiency and other disorders, such as seizures, learning difficulties, visual and hearing impairment, behavioral deficits, minimal brain dysfunction syndrome, delay of neuro-psycho-motor development, hypoxic-ischemic encephalopathy, and cerebral palsy (3-6). An estimated 23% of worldwide deaths per year during the first four weeks of life are due to NA and the consequences of hypoxic-ischemic insults affect 2-4 newborns (NB) per 1,000 live births. Among premature babies, the death rate due to NA increases to about 60% (7) and can cause necrotizing enterocolitis (NEC) in 6% of them (8-10).

Caspase-3 expression determines the apoptotic level of the cell and can be considered as a marker for inflammation in diseases that cause brain damage, such as Parkinson's (11). Similarly, I-FABP (intestinal fatty acid-binding protein) is considered a marker for intestinal damage, such as in necrotizing enterocolitis (12).

Studies in animal models of neonatal asphyxia, hypoxia, ischemia/reperfusion and NEC show changes in the expression of caspase-3 and I-FABP markers (12,13). However, these findings were not correlated with simultaneous brain and intestinal damage. Our aim was to investigate the effect of ischemia and reperfusion after mechanical ventilation in the brain and intestine of premature and term rat fetuses that underwent neonatal asphyxia, using the caspase-3 and I-FABP as markers.

## Material and Methods

The project was approved by the Ethics Committee of Animal Experimentation (CETEA) of the Faculdade de Medicina de Ribeirão Preto, Universidade de São Paulo, Ribeirão Preto, SP, Brazil (#040/2011).

### Experimental groups and sample collection

Pregnant Sprague-Dawley dams were divided into two groups: preterm (PT) delivery, in which the pregnancy was terminated at 20.5 days of gestation (DG), and term (T) delivery, in which the pregnancy was terminated at 21.5 DG (term=22 days). We decided to ventilate at 20.5 DG because of the feasibility to perform endotracheal intubation and also due to the equivalence of 30 weeks of human gestational age (14).

Newborn pups were divided into eight subgroups (n=12/group): 1) preterm control (PTC), 2) preterm ventilated (PTV), 3) preterm asphyxiated (PTA), 4) preterm asphyxiated and ventilated (PTAV), 5) term control (TC), 6) term ventilated (TV), 7) term asphyxiated (TA), and 8) term asphyxiated and ventilated (TAV). The dams were anesthetized with an intramuscular injection of 50 mg/mL ketamine in combination with 10 mg/mL xylazine and submitted to laparotomy. The fetuses were removed from the uterus and weighed.

The newborn pups were sacrificed by decapitation soon after the ventilation and/or asphyxia procedure. The control groups (PTC and TC) were sacrificed immediately after delivery. The brain and 2-3 cm long fragments of the proximal jejunum and distal ileum were collected and fixed in 10% formaldehyde. The full intestine was removed and frozen in liquid nitrogen for the molecular biology study.

### Induction of asphyxia

Asphyxia was induced according to the model described by Takada et al. (15). The animals of the PTA, PTAV, TA, and TAV subgroups were removed from the uterus in doubles and positioned in an acrylic anoxic chamber with a lid (dimensions: 30×20×12.5 cm) and maintained in a water bath for temperature control (37-38°C). The chamber was filled with nitrogen (N_2_) at a flow of 5 L/min for a period of 30 min.

### Pulmonary ventilation

The PTV, PTAV, TV, and TAV subgroups were ventilated with a Mini-Vent type 845 mechanical mini-ventilator (Harvard Apparatus^®^, Hugo Sachs Eletronik Harvard Apparatus GmbH, Germany), according to Gallindo et al. (16). The newborn pups were positioned on a heated table where they were intubated with an intravascular teflon 24G Vialon™ catheter (BD Insyte Autoguard, Becton Dickinson Infusion Therapy System Inc., USA) utilizing a surgical microscope with 4.5× magnification (DFV, Vasconcellos, Brazil), with a continuous 100% oxygen (O_2_) flow with a cycling frequency of 80 rpm, FiO_2_ of 1.0, inspiratory/expiratory ratio of 1:1, and positive end expiratory pressure of 0 cmH_2_O, for 30 min. The ventilatory volume was 50 µL/g with a frequency of 80/min in the PTV and PTAV subgroups and 75 µL/g with the same frequency in the TV and TAV subgroups.

### Morphological evaluation and histological processing

Body weight (BW), brain weight (BrW), brain/body weight ratio (BrW/BW), intestinal weight (IW) and the intestinal/body weight ratio (IW/BW) were measured. In addition, the brain and intestinal segments (jejunum and ileum) were dehydrated in increasing ethanol series, cleared with xylene and embedded in histological paraffin. Five-micrometers transverse sections of brain (dorsal cortex and hippocampus), intestine, jejunum and ileum were stained with Masson trichrome or submitted to immunohistochemistry (IHC). The brain histological sections were photographed with an AxiosKop2 plus microscope and AxioCam Hrc (Carl Zeiss Microscopy GmbH, Germany) using Axio Vision 3.1 software at 40× magnification. The intestine sections were photographed with a NIKON Eclipse E200 80*i* photomicroscope (Nikon, Japan) at 200× magnification, and the images analyzed. The IHC slides were analyzed by three examiners who individually assigned arbitrary values (av) according to the intensity of the immunostaining and a mean score was then calculated for each section.

### Histological grading of brain and intestinal injury

The brain histology slides were evaluated by three independent reviewers. General tissue architecture and structure, and cellular density of 16 brain segments, divided into cortex and hippocampus (2 fetuses/group), were analyzed.

For the intestinal evaluation, three jejunum and ileum segments were obtained from each animal (96 sections of 4 fetuses/group). The slides were analyzed by three independent reviewers, and graded according to Dvorak et al. (17): 0 = no damage; 1 = slight separation of the lamina propria and/or submucosa; 2 = moderate separation of the lamina propria and/or submucosa and/or edema of the submucosal and muscular layers; 3 = severe separation of the lamina propria and/or submucosa and/or severe edema of the submucosal and muscular layers, and/or desquamation of the villi; 4 = loss of villi and necrosis. Intermediate scores of 0.5, 1.5, 2.5 and 3.5 were used for a more precise assessment of intestinal damage level. Significant tissue involvement was considered to be present in animals with a histological score ≥2.

### Caspase-3 and I-FABP immunohistochemical analysis

Transverse sections brain slides (dorsal cortex and hippocampus) were selected for IHC. Endogenous peroxidase was blocked by incubating slides with 30% H_2_O_2_ and 3% methanol for 10 min. Antigen exposure was performed in vapor for 40 min in 50 mM Tris-HCl buffer, pH 9.5. Sections were blocked with 10% rabbit serum in PBS, pH 7.4, for 30 min, incubated overnight at 4^o^C with the primary antibody (anti-caspase-3 sc-7148 and anti-I-FABP sc-16063, Santa Cruz Biotechnology, USA) diluted 1:100 and 1:200, respectively, in BSA 3%. Sections were washed in PBS with 2% Triton X-100, pH 7.4, for 20 min and the secondary antibodies (sc-2040 and sc-2768, Santa Cruz Biotechnology) diluted 1:300 and 1:200 in BSA 3% respectively, was added for 2 h. Avidin-biotin (Vectastain ABC kit, Vector Labs, USA) was diluted 1:20 in PBS plus Tween, pH 7.4, for 30 min then developed with a solution of 3,3′-diaminobenzidine-tetra-hydrochloride (Sigma, USA) and 0.03% hydrogen peroxide in 50 mM PBS, pH 7.6, for 5 min. Sections were counterstained with Harris hematoxylin, dehydrated and mounted with Permount^®^ (Fisher Scientific, USA). Caspase-3 was scored by counting the positively-stained cells in all hippocampal and brain dorsal cortex areas. The intensity of I-FAPB staining was scored as follows: 0 = negative, 1 = weak, 2 = moderate, 3 = deep, and 4 = very deep staining. Intermediate scores of 0.5, 1.5, 2.5 and 3.5 were also used for a more precise assessment of tissue staining levels.

### I-FABP expression by Western blot analysis

Six intestines per subgroup were homogenized in an extraction buffer and centrifuged in a Mikro 200R centrifuge (Hettich, Germany) at 13.684 *g* at 4°C for 30 min, and protein concentration determined using the Bradford method. Protein aliquots of 20 µg were separated by SDS-PAGE under a constant current (100 V) for 2 h and transferred to a nitrocellulose membrane at 120 V for 90 min at 4°C. Blots were blocked with skim milk and constant shaking and membranes were incubated overnight at 4°C with anti-I-FABP primary antibody (sc-16063, Santa Cruz Biotechnology) diluted 1:100 in 3% PBS/BSA. On the subsequent day, the membranes were washed with 0.01 M PBS buffer, pH 7.4, and incubated with secondary antibody (sc-2768, Santa Cruz Biotechnology) diluted 1:2000 in 3% PBS/BSA for 2 h. The membranes were washed, and a chemiluminescence kit was used (Pierce, USA) for visualization. Blots were developed and photographed using the ChemiDoc XRS+ with Image Lab Software (Bio-Rad Laboratories, USA).

### Tissue malondialdehyde (MDA)

To assess the oxidative stress, brain and intestine MDA levels were measured in 4 animals from each group. Protein extraction was performed with the same protocol as used for western blot. We determined tissue MDA spectrophotometrically at 532 nm using 1,1,3,3-tetramethoxypropan (Sigma-Aldrich, USA) as the standard, according to Ohkawa et al. (18). The values were reported as µm/mg protein.

### Statistical analysis

Morphometry and western blot data were analyzed statistically by ANOVA followed by the Tukey-Kramer post-test. The IHC scores for both markers were analyzed by the Kruskal-Wallis test followed by the Dunn post-test. The level of significance was set at P<0.05 in all analyses. The calculations were made using the GraphPadPrism 3.02 software (GraphPad Software Inc., USA).

## Results

None of the ventilated groups (PTV, PTAV, TV and TAV) had complications during the ventilation procedure.

### Morphological analysis

BW, BrW, IW, BrW/BW, and IW/BW, and intestinal tissue damage scores are shown in Table 1. BW, BrW, and IW were lower in the preterm compared to the term groups. The IW was lower in the TA subgroup in comparison to the others term groups (TC, TV, and TAV) (P<0.05) and the IW/BW ratio was lower in the TA than in the TAV subgroup (P<0.005). There were no differences in BrW and BrW/BW between groups (Table 1).

**Table:**
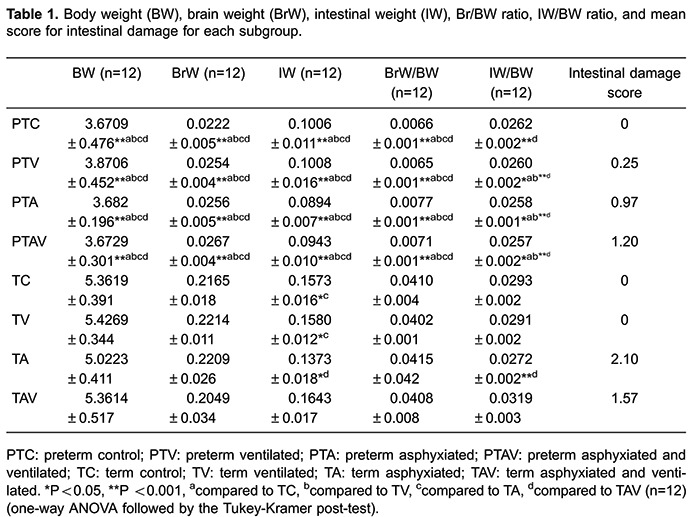

### Histological grading of brain injury

**Figure 1:**
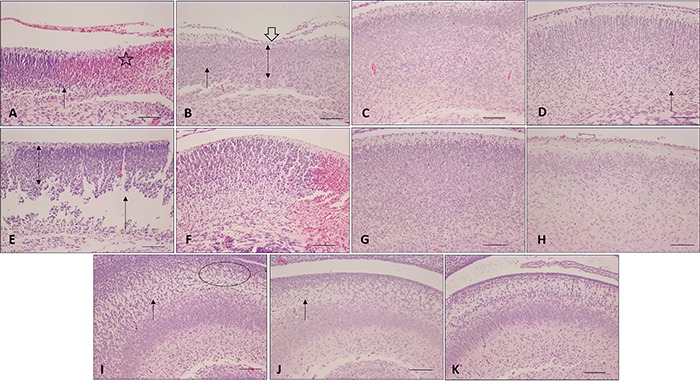
Photomicrographs of histological slides of newborn rat brains. Cerebral cortex: *A*: preterm asphyxiated rats; *B*: preterm asphyxiated and ventilated rats; *C*: preterm control rats; *D*: preterm ventilated rats; *E*: term asphyxiated rats; *F*: term asphyxiated and ventilated rats; *G*: term control rats; *H*: term ventilated rats. Hippocampus: *I:* preterm asphyxiated rats; *J*: preterm asphyxiated and ventilated rats; *K*: preterm control rats. Note the mild cortical disorganization (arrows), vacuolization area (circles), midbrain bleeding (star), reduction of cortical volume (double arrows), reduction of molecular layer, scar injury extended from the dorsal cortex (open arrow), with loss of gray matter areas. Hematoxylin and eosin. Magnification: 10×; scale bar: 100 μm.

PTC, TC, and TV subgroups demonstrated preservation of cortical lamination and no brain lesions. The PTV subgroup presented a mild cortical disorganization. The PTA and PTAV subgroups presented high levels of injury. In the PTA subgroup, these injuries were vacuolization areas, cortical bleeding, and impaired cortical and hippocampus lamination. The PTAV subgroup showed cortical, hippocampus and midbrain bleeding, reduction of cortical volume, impaired lamination, absence of molecular layer, damage in hippocampus, and scar injury extended from the dorsal cortex to the lateral ventricles in the frontal lobe. The TA subgroup presented cortical disorganization, impaired tissue areas and reduction of the cortical thickness. TAV presented cortical disorganization and areas of gray matter loss. There was no difference in hippocampus in group TA and TAV (Figure 1).

### Histological grading of the intestinal injury

**Figure 2:**
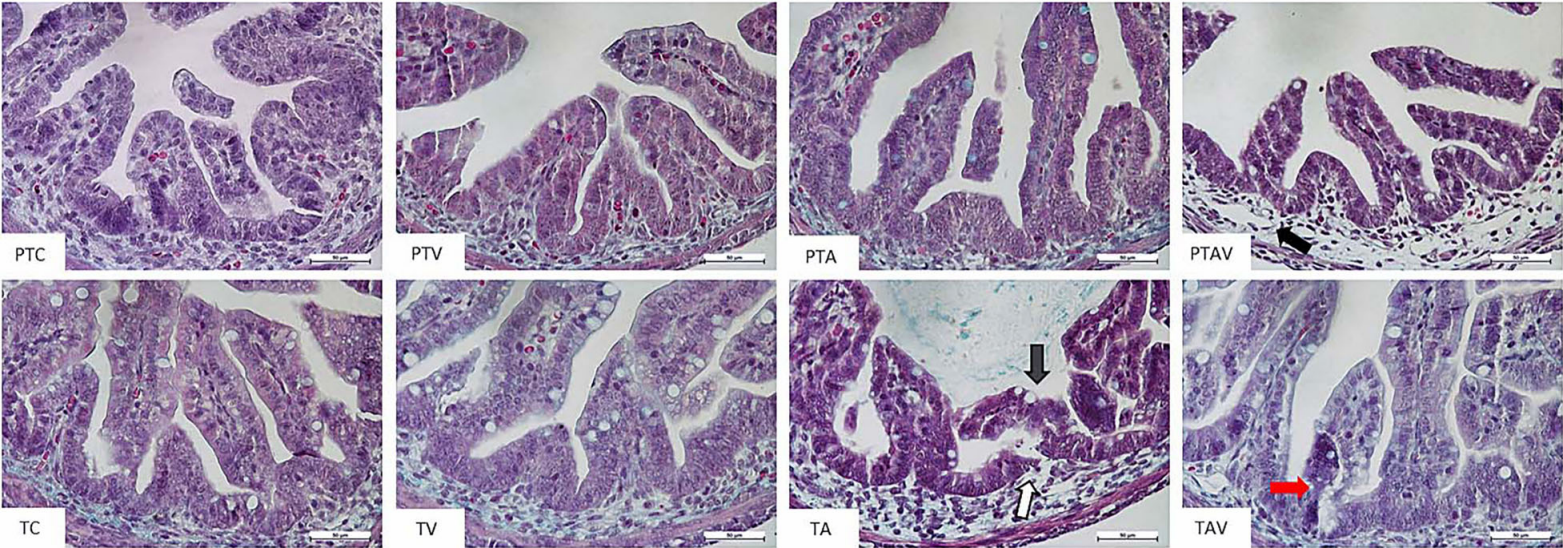
Masson's trichrome staining of collected intestines of newborn rats. Observe slight separation of the lamina propria in the preterm asphyxiated/ventilated rats (PTAV) (score = 1.25; black arrow), desquamation of the villi (gray arrow) and moderate separation of lamina propria in term asphyxiated rats (TA) (score=2.10; white arrow), and slight separation of the lamina propria in term asphyxiated and ventilated rats (TAV) (score=1.57) (red arrow); n=4. PTC: preterm control; PTV: preterm ventilated; PTA: preterm asphyxiated; TC: term control; TV: term ventilated. Magnification: 200×; scale bar: 50 μm.

The TA subgroup was the only one that showed intestinal damage with a score higher than 2, which is indicative of NEC. However, the PTAV and TAV subgroups had a score ranging from 1 to 1.5, indicating structural changes (Figure 2).

### Caspase-3 immunohistochemical analysis

**Figure 3:**
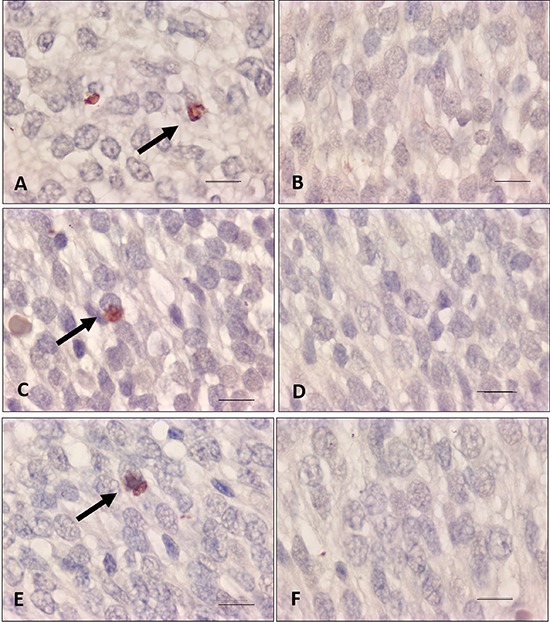
. Photomicrograph of newborn rat brain immunostained for caspase-3. Cerebral cortex: *A*: preterm asphyxiated and ventilated (PTAV); *B*: preterm control (PTC); *C*: term asphyxiated (TA); *D*: term control (TC). Hippocampus: *E*: TA; *F*: TC. In the preterm group, caspase-3 expression was significantly higher in the cortex area of the PTAV compared with the PTC subgroup (P<0.05). In the term group, the caspase-3 expression was higher in the cortex and hippocampus areas of the TA compared with the TC subgroup. Note intensely marked astrocytes with hypertrophic processes (arrows). Magnification: 100× (oil immersion); scale bar: 10 μm.

In the preterm group, caspase-3 expression was significantly higher in the cortex area of PTAV subgroup compared with PTC subgroup (P<0.05). In the term group, the caspase-3 expression was higher in the cortex and hippocampus areas of the TA subgroup compared with the TC subgroup (P<0.005; Figure 3). There was no statistical difference in caspase-3 expression among the other groups (PTV, PTA, TV, and TAV).

### I-FABP immunohistochemical analysis

**Figure 4:**
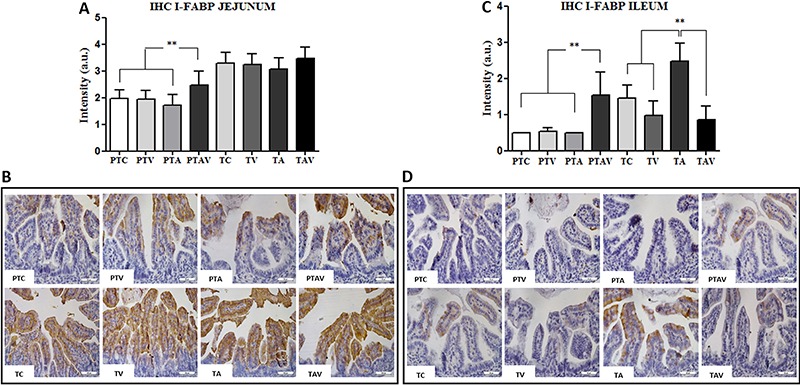
Immunohistochemistry (IHC) results of the fatty acid-binding intestinal protein (I-FABP) expression in newborn rats. *A*, IHC arbitrary units (a.u.) of I-FABP in the jejunum. *B*, IHC photomicrographs of jejunum. Observe the increased I-FABP immunostaining in the PTAV subgroup compared to all PT subgroups. *C*, IHC arbitrary units (a.u.) of the I-FABP expression in the ileum. *D*, IHC photomicrographs of ileum. Observe increased I-FABP immunostaining in the PTAV subgroup compared to all PT subgroups (top images), and increased I-FABP immunostaining in TA subgroup compared to all T subgroups (bottom images). PTC: preterm control; PTV: preterm ventilated; PTA: preterm asphyxiated; PTAV: preterm asphyxiated and ventilated; TC: term control; TV: term ventilated; TA: term asphyxiated; TAV: term asphyxiated and ventilated. Magnification: 200 ×; scale bar: 50 μm; n=4. **P<0.005, Kruskal-Wallis test followed by the Dunn post-test.

In the preterm group, I-FABP expression was higher in the jejunum and ileum of the PTAV subgroup, compared with the other subgroups (P<0.0001). In the term group, I-FABP expression was higher in the ileum of the TA subgroup compared with all subgroups (P<0.05; Figure 4).

### I-FABP expression by western blot analysis

**Figure 5:**
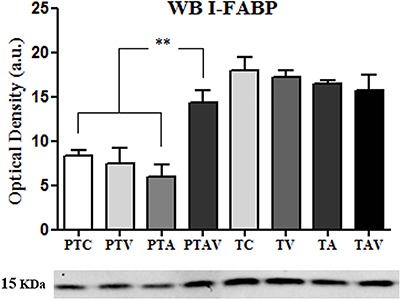
Expression of fatty acid-binding intestinal protein (I-FABP) by optical density (arbitrary units, a.u.) after western blotting in newborn rats (n=6). Observe higher expression in the term subgroups compared to the preterm subgroups, and the increased expression in the PTAV subgroup compared to the other preterm subgroups. PTC: preterm control; PTV: preterm ventilated; PTA: preterm asphyxiated; PTAV: preterm asphyxiated and ventilated; TC: term control; TV: term ventilated; TA: term asphyxiated; TAV: term asphyxiated and ventilated. **P<0.005, one-way ANOVA followed by the Tukey-Kramer post-test.

In the preterm group, I-FABP expression was increased in the PTAV subgroup (P<0.0001). There was no significant difference among the subgroups of the term group (Figure 5).

### Tissue MDA

**Figure 6:**
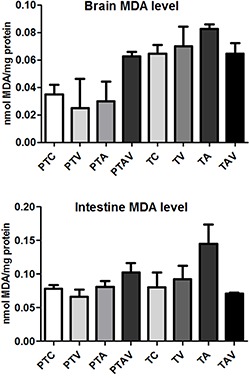
Brain and intestine malondialdehyde (MDA) levels in newborn rats. PTC: preterm control; PTV: preterm ventilated; PTA: preterm asphyxiated; PTAV: preterm asphyxiated and ventilated; TC: term control; TV: term ventilated; TA: term asphyxiated; TAV: term asphyxiated and ventilated.

The brain and intestine MDA levels were increased in PTAV and TA. However, they did not show significant differences (Figure 6).

## Discussion

The main therapeutic measure used over the past century for the care of asphyxiated NB is immediate oxygen support by endotracheal intubation and/or mechanical ventilation. However, the indiscriminate use of this intervention may be potentially toxic to various organs of the NB, resulting in serious diseases and intensifying the development of injuries (18). After asphyxia, during the reperfusion period, molecular oxygen in the tissue reacts with hypoxanthine and triggers production of free radical species causing tissue damage (19-21).

There was no difference in any of the subgroups in relation to BW, BrW and BrW/BW ratio. TA subgroup showed lower IW and IW/BW compared to all other term subgroups. This result could be explained by the severity of the injury induced by asphyxia and were similar to previous publications that obtained decreased BW neonates in the model of perinatal and intrauterine asphyxia (22,23).

Brain lesions were found on histological analysis in the asphyxiated (PTA and TA) and ventilated (PTAV and TAV) subgroups. However, only the PTAV and TA subgroups presented severe structural disorganization of the cortex and hippocampus, such as volume reduction and presence of bleeding. Although brain damage is related to the duration and severity of hypoxia, it is also a result of a complex cascade of secondary factors in the post-hypoxia period or during the re-oxygenation of tissue, resulting in disruption of ionic homeostasis and consequent failure of the ionic flow through the cell membrane (24).

Apoptosis is a prominent form of neuronal death in the neonatal hypoxia/ischemia model. In cerebral ischemia, the caspase cascade is mediated specially by caspase-3, which is one of the 10 sub-caspase family members, classified as an executioner subclass member. Activated directly by caspase-8, caspase-3 stimulates the mitochondrial pathway causing apoptosis, and it is considered a biomarker of neuronal apoptosis and of brain lesion levels (13,25).

In our study, the expression of caspase-3 in the PTAV subgroup was higher compared to PTC in the cerebral cortex, but was not different in the hippocampus. The PTA subgroup presented brain lesion but did not present changes in caspase-3 staining. In the term group, the TA subgroup presented higher expression compared to TC in both the cerebral cortex and hippocampus. The increase of caspase-3 expression in the brain has been reported in neonatal asphyxia (26). Our results were similar to the ones by Yang et al. (27), who demonstrated that peripartal ischemia can induce neuronal apoptosis after birth. In that study, caspase-3 positive-neurons and TUNEL staining were increased in the hippocampus of rats submitted to perinatal asphyxia (10-15 min) on postnatal days 1, 3, and 7. Hattori et al. (28) administered blood cells from umbilical cord intraperitoneally after hypoxic-ischemic brain injury in a neonatal rat model and found antiapoptotic and antioxidative effects 24 h later, demonstrated by a decrease in the number of cells positive for active caspase-3. Huang et al. (29) reported increased levels of the cleaved caspase-3 protein in the brain of preterm newborns rats in day 1 of a time-dependent hypoxic-ischemic induced encephalopathy.

The results of the histological analysis of the intestinal tissues were similar to those of the brain. The PTAV and TA subgroups had higher injury scores than the other groups. Similar results were reported by Varga et al. (30) who detected moderate injuries in the intestine of adult rats after 1 h of ischemia followed by reperfusion for 0 to 24 h. In perinatal asphyxia performed in neonatal pigs submitted to hypoxia (10-15% O_2_) followed by resuscitation, a high degree of ileum injury, similar to NEC, was found. These injuries were explained by the increased lactate secondary to metabolic acidosis during the hypoxia (31,32).

Few reports are available in the literature about ischemia/reperfusion damage to the intestine of newborn rats. Thus, a comparison of the ischemia results obtained in the present study is limited. However, some evaluations of the histological injury caused by ischemia/reperfusion in the perinatal period were found. Xu et al. (22) detected hyperemia and separation of the lamina propria in the intestine of 24-hours old rats whose dams had been submitted to clamping of the uterine vessels for 20 minutes. Meyer et al. (33) detected an increased injury score in the intestine of rats submitted to ischemia/reperfusion up to the fourth day of life.

Cross et al. (34) reported that 5% of the oxygen consumed by the cells is metabolized in the form of ROS (reactive oxygen species): superoxide radical (O_2_•−), hydrogen peroxide (H_2_O_2_), and hydroxyl radical (OH•). When ischemia/reperfusion occurs, there is a change in the enzymatic reaction of the respiratory chain. The hypoxanthine cascade occurs at the beginning of hypoxia, followed by a reaction between superoxide and hydrogen peroxide radicals (O_2_•− and H_2_O_2_) during reperfusion, resulting in the production of hydroxyl radicals (OH•).

The hydroxyl radical (OH•) is highly reactive and therefore is the main factor responsible for direct cell injuries, such as damage to the lipoprotein membrane and its intracellular components, changes in permeability and in protein, nucleic acid, lipid and carbohydrate structure (35). To fight the damage generated by ROS the organism counts on antioxidant enzymes such as the superoxide dismutases, catalases and glutathione peroxidases. However, studies have confirmed that this metabolic response of the antioxidant defense system is proportional to the age of the organism (36,37). Our histological results can be explained by the reactions that generate ROS *versus* antioxidants. In spite of increasing, the brain and intestine MDA levels in PTAV and TA did not show significant differences. Possibly, the PTAV subgroup showed higher levels of histological injury due to the low amounts of antioxidants, being more affected during the reoxygenation than the asphyxia period. In contrast, the older rats of the TA subgroup showed higher levels of injury during the ischemia (P<0.05), while the TAV subgroup had the injury level reversed when reoxygenated.

I-FABP protein is a member of a family of 9 FABPs. Its expression is abundant in the cytoplasm of epithelial cells of the small intestine and can be easily released by epithelial cells into the bloodstream when the tissue is injured. I-FABP is used as biomarker for NEC, and can also indicate intestinal injury during the early stages of disease. The treatment of experimental NEC in rats with probiotics decrease the expression of I-FABP (12). In our study, the expression of I-FABP in the preterm group was increased in the jejunum and ileum of PTAV subgroup, and in the term group was increased only in the ileum of TA subgroup.

The relationship between I-FABP expression in plasma and jejunum of elderly patients undergoing pancreaticoduodenectomy surgery was evaluated by Schellekens et al. (38). They found that the duration of ischemia and the extension of intestinal damage were related to the increase of I-FABP in plasma levels and the intense staining in subepithelial space after 30 min of ischemia with disruption of the epithelium in the immunohistochemistry. Despite these findings, there was no difference in I-FABP levels after the reperfusion time of 30 and 120 min. The same was found in the model of intestinal ischemia in pigs and the NEC model in rats. The I-FABP increased proportionally to the time of injury, while at the tissue level the increase occurred after 30 min of ischemia (39,40).

Finally, the effects of ischemia and reperfusion utilizing mechanical ventilation had different responses in preterm and term newborn pups exposed to neonatal asphyxia. There was an increase of caspase-3 and I-FABP expression. In the term pups, there was an increased expression of both markers in the TA subgroup. Our research demonstrates that preterm newborn pups were more susceptible to brain and intestinal injuries generated by reperfusion, and term pups had a greater susceptibility to the enzymatic reactions occurring during ischemia. Some limitations must be considered in this study, such as limited access to intestinal injury biomarkers in plasma levels, and quality of ROS markers. However, these experimental results support the concept of different brain and intestinal responses caused by neonatal asphyxia according to gestational age and may hold encouraging prospects for neonatal clinical care.

## References

[1] Martin RJ, Wang K, Koroglu O, Di Fiore J, Kc P (2011). Intermittent hypoxic episodes in preterm infants: do they
matter?. Neonatology.

[2] Hall DM (1989). Birth asphyxia and cerebral palsy. BMJ.

[3] Frizzo JK, Cardoso MP, de Assis AM, Perry ML, Volonte C, Frizzo ME (2010). Effects of acute perinatal asphyxia in the rat
hippocampus. Cell Mol Neurobiol.

[B04] Azra HB, Bhutta ZA (2006). Birth asphyxia in developing countries: current status and public
health implications. Curr Probl Pediatr Adolesc Health Care.

[B05] Calkavur S, Akisu M, Olukman O, Balim Z, Berdeli A, Cakmak B (2011). Genetic factors that influence short-term neurodevelopmental outcome
in term hypoxic-ischaemic encephalopathic neonates. J Int Med Res.

[6] Coq JO, Strata F, Russier M, Safadi FF, Merzenich MM, Byl NN (2008). Impact of neonatal asphyxia and hind limb immobilization on
musculoskeletal tissues and S1 map organization: implications for cerebral
palsy. Exp Neurol.

[7] Reddy NR, Krishnamurthy S, Chourasia TK, Kumar A, Joy KP (2011). Glutamate antagonism fails to reverse mitochondrial dysfunction in
late phase of experimental neonatal asphyxia in rats. Neurochem Int.

[8] Schnabl KL, Van Aerde JE, Thomson AB, Clandinin MT (2008). Necrotizing enterocolitis: a multifactorial disease with no
cure. World J Gastroenterol.

[B09] Torrazza RM, Li N, Neu J (2014). Decoding the enigma of necrotizing enterocolitis in premature
infants. Pathophysiology.

[10] DeMauro SB, Roberts RS, Davis P, Alvaro R, Bairam A, Schmidt B (2011). Impact of delivery room resuscitation on outcomes up to 18 months in
very low birth weight infants. J Pediatr.

[11] Macchi B, Di Paola R, Marino-Merlo F, Felice MR, Cuzzocrea S, Mastino A (2015). Inflammatory and cell death pathways in brain and peripheral blood in
Parkinson's disease. CNS Neurol Disord Drug Targets.

[12] Goncalves FL, Soares LM, Figueira RL, Simoes AL, Gallindo RM, Sbragia L (2015). Evaluation of the expression of I-FABP and L-FABP in a necrotizing
enterocolitis model after the use of *Lactobacillus acidophilus*. J Pediatr Surg.

[13] Zhang F, Yin W, Chen J (2004). Apoptosis in cerebral ischemia: executional and regulatory signaling
mechanisms. Neurol Res.

[14] Toelen J, Carlon M, Claus F, Gijsbers R, Sandaite I, Dierickx K (2011). The fetal respiratory system as target for antenatal
therapy. Facts Views Vis Obgyn.

[15] Takada SH, Sampaio CA, Allemandi W, Ito PH, Takase LF, Nogueira MI (2011). A modified rat model of neonatal anoxia: Development and evaluation by
pulseoximetry, arterial gasometry and Fos immunoreactivity. J Neurosci Methods.

[16] Gallindo RM, Goncalves FL, Figueira RL, Simoes AL, Sbragia L (2014). Standardization of pulmonary ventilation technique using
volume-controlled ventilators in rats with congenital diaphragmatic
hernia. Rev Col Bras Cir.

[17] Dvorak B, Halpern MD, Holubec H, Dvorakova K, Dominguez JA, Williams CS (2003). Maternal milk reduces severity of necrotizing enterocolitis and
increases intestinal IL-10 in a neonatal rat model. Pediatr Res.

[18] Ohkawa H, Ohishi N, Yagi K (1979). Assay for lipid peroxides in animal tissues by thiobarbituric acid
reaction. Anal Biochem.

[19] Gane B, Bhat BV, Adhisivam B, Joy R, Prasadkumar P, Femitha P (2014). Risk factors and outcome in neonatal necrotising
enterocolitis. Indian J Pediatr.

[B20] Maretta M, Toth S, Bujdos M, Toth S, Jonecova Z, Vesela J (2012). Alterations of epithelial layer after ischemic preconditioning of
small intestine in rats. J Mol Histol.

[21] Hua S, Zhang X, Zhang S, Xu J, Feng Z (2012). Effects of different ventilation strategies on lung injury in newborn
rabbits. Pediatr Pulmonol.

[22] Xu L, Dong W, Zhao J, Xu Y (2015). Effect of Marine collagen peptides on physiological and
neurobehavioral development of male rats with perinatal asphyxia. Mar Drugs.

[23] Cox-Limpens KE, Strackx E, Van den Hove DL, Van Ekkendonk, Jong M, Zimmermann LJ (2015). Fetal asphyctic preconditioning protects against perinatal
asphyxia-induced apoptosis and astrogliosis in neonatal brain. CNS Neurol Disord Drug Targets.

[24] Van Reempts J (1984). The hypoxic brain: histological and ultrastructural
aspects. Behav Brain Res.

[25] Cohen GM (1997). Caspases: the executioners of apoptosis. Biochem J.

[26] Sugawara T, Noshita N, Lewen A, Gasche Y, Ferrand-Drake M, Fujimura M (2002). Overexpression of copper/zinc superoxide dismutase in transgenic rats
protects vulnerable neurons against ischemic damage by blocking the mitochondrial
pathway of caspase activation. J Neurosci.

[27] Yang T, Zhuang L, Terrando N, Wu X, Jonhson MR, Maze M (2011). A clinically relevant model of perinatal global ischemic brain damage
in rats. Brain Res.

[28] Hattori T, Sato Y, Kondo T, Ichinohashi Y, Sugiyama Y, Yamamoto M (2015). Administration of umbilical cord blood cells transiently decreased
hypoxic-ischemic brain injury in neonatal rats. Dev Neurosci.

[29] Huang Y, Lai H, Xu H, Wu W, Lai X, Ho G (2013). Impact of perinatal systemic hypoxic-ischemic injury on the brain of
male offspring rats: an improved model of neonatal hypoxic-ischemic encephalopathy
in early preterm newborns. PLoS One.

[30] Varga J, Toth S, Toth S, Tomeckova V, Gregova K, Vesela J (2012). The relationship between morphology and disaccharidase activity in
ischemia-reperfusion injured intestine. Acta Biochim Pol.

[31] Gill RS, Lee TF, Sergi C, Bigam DL, Cheung PY (2012). Early versus delayed cyclosporine treatment in cardiac recovery and
intestinal injury during resuscitation of asphyxiated newborn
piglets. Intensive Care Med.

[32] Young CM, Kingma SD, Neu J (2011). Ischemia-reperfusion and neonatal intestinal injury. J Pediatr.

[33] Meyer KF, Martins JL, Freitas LG, Oliva ML, Patricio FR, Macedo M (2006). [Evaluation of an experimental model of necrotizing enterocolitis in
rats]. Acta Cir Bras.

[34] Cross CE, Halliwell B, Borish ET, Pryor WA, Ames BN, Saul RL (1987). Oxygen radicals and human disease. Ann Intern Med.

[35] Heffner JE, Repine JE (1989). Pulmonary strategies of antioxidant defense. Am Rev Respir Dis.

[36] Frank L, Sosenko IR (1987). Development of lung antioxidant enzyme system in late gestation:
possible implications for the prematurely born infant. J Pediatr.

[37] Lindeman JH, van Zoeren-Grobben D, Schrijver J, Speek AJ, Poorthuis BJ, Berger HM (1989). The total free radical trapping ability of cord blood plasma in
preterm and term babies. Pediatr Res.

[38] Schellekens DH, Grootjans J, Dello SA, van Bijnen AA, van Dam RM, Dejong CH (2014). Plasma intestinal fatty acid-binding protein levels correlate with
morphologic epithelial intestinal damage in a human translational
ischemia-reperfusion model. J Clin Gastroenterol.

[39] Mitidiero LF, Simoes AL, Goncalves FL, Figueira RR, Castro e Silva, Sbragia L (2014). L-FABP and I-FABP expression in newborn rats changes inversely in the
model of necrotizing enterocolitis. Acta Cir Bras.

[40] Kano H, Okada K, Morimoto K, Bao W, Fukase K, Ito A (2015). Prediction of reversibility of intestinal mucosal damage after
ischemia-reperfusion injury by plasma intestinal fatty acid-binding protein levels
in pigs. Perfusion.

